# A phase I trial of NK-92 cells for refractory hematological malignancies relapsing after autologous hematopoietic cell transplantation shows safety and evidence of efficacy

**DOI:** 10.18632/oncotarget.19204

**Published:** 2017-07-12

**Authors:** Brent A. Williams, Arjun Datt Law, Bertrand Routy, Neal denHollander, Vikas Gupta, Xing-Hua Wang, Amélie Chaboureau, Sowmya Viswanathan, Armand Keating

**Affiliations:** ^1^ Cell Therapy Program, Princess Margaret Cancer Centre, Toronto, ON, Canada; ^2^ Department of Laboratory Medicine, University Health Network, Toronto, ON, Canada

**Keywords:** NK cell, NK-92, clinical trial, lymphoma, multiple myeloma

## Abstract

**Background:**

Autologous NK cell therapy can treat a variety of malignancies, but is limited by patient-specific variations in potency and cell number expansion. In contrast, allogeneic NK cell lines can overcome many of these limitations. Cells from the permanent NK-92 line are constitutively activated, lack inhibitory receptors and appear to be safe based on two prior phase I trials.

**Materials and Methods:**

We conducted a single-center, non-randomized, non-blinded, open-label, Phase I dose-escalation trial of irradiated NK-92 cells in adults with refractory hematological malignancies who relapsed after autologous hematopoietic cell transplantation (AHCT). The objectives were to determine safety, feasibility and evidence of activity. Patients were treated at one of three dose levels (1 × 10^9^ cells/m^2^, 3 × 10^9^ cells/m^2^ and 5 × 10^9^ cells/m^2^), given on day 1, 3 and 5 for a planned total of six monthly cycles.

**Results:**

Twelve patients with lymphoma or multiple myeloma who relapsed after AHCT for relapsed/refractory disease were enrolled in this trial. The treatment was well tolerated, with minor toxicities restricted to acute infusional events, including fever, chills, nausea and fatigue. Two patients achieved a complete response (Hodgkin lymphoma and multiple myeloma), two patients had minor responses and one had clinical improvement on the trial.

**Conclusions:**

Irradiated NK-92 cells can be administered at very high doses with minimal toxicity in patients with refractory blood cancers, who had relapsed after AHCT. We conclude that high dose NK-92 therapy is safe, shows some evidence of efficacy in patients with refractory blood cancers and warrants further clinical investigation.

## INTRODUCTION

The therapy of aggressive hematological cancers relapsing after autologous hematopoietic cell transplantation [AHCT] is particularly challenging and sustained remissions are rare. While allogeneic HCT offers the possibility of cure, albeit low, treatment-related morbidity and mortality exert a high toll [1]. Adoptive immunotherapy may provide an acceptable alternative, although adverse effects with T cell therapy can be serious and even fatal [2–5]. While emerging data suggest that NK cell therapy may be a less toxic alternative, results with autologous NK cells have been disappointing and outcomes with primary allogeneic NK cells are variable (reviewed in [6]). Limitations of autologous primary NK cells include patient variability in cell number and function, inhibition of cytotoxicity by tumors expressing self-HLA that engage inhibitory killer immunoglobulin-like receptors, inhibition by the tumour microenvironment and challenges in expanding sufficient numbers of cells [7, 8]. A potentially attractive alternative that overcomes many of these limitations is the use of permanent NK cell lines. NK-92 is the only malignant NK cell line to have been investigated in clinical trials [9, 10], is IL-2 dependent, was derived from a patient with a NK-cell non-Hodgkin lymphoma with a CD56+CD3-CD16-immunophenotype and retains cytotoxic anti-tumor activity [11]. Its advantages include a high degree of cytotoxicity, less variability in cytotoxic potency, limited inhibitory receptor expression and near-unlimited expansion capacity [12]. NK-92 has been evaluated in clinical trials for renal cell carcinoma, melanoma[9] and solid tumors and leukemia/lymphoma[10]. Minimal toxicities were noted despite the infusion of large numbers of NK-92 cells.

Here, we report a Phase I, dose escalation trial of NK-92 to assess safety and preliminary evidence of efficacy in 12 patients with relapsed, refractory hematological malignancies whose disease recurred after AHCT. Patients received up to 6 monthly cycles of irradiated NK-92. Toxicity was minimal and 5 of the 12 patients responded, including one long term survivor in remission for 10 years.

## RESULTS

### Patients

Patients (*n* = 12) with relapsed/refractory hematological malignancies who relapsed after AHCT were enrolled into this study between 2005 and 2015. Their characteristics are summarized in Table 1. Three females and nine males with a median age of 59 years (range, 42–67 years) received standard of care with salvage therapy followed by AHCT for multiple myeloma (*n* = 5), Hodgkin lymphoma (*n* = 2) and non-Hodgkin lymphoma (*n* = 5). All patients had been treated with conventional chemotherapy as per standard of care at the time of initial diagnosis. Two patients with diffuse large B cell lymphoma (DLBCL) received Rituximab as part of their chemotherapy protocols. Patients with multiple myeloma received multiple agents including bortezomib, lenalidomide and thalidomide. All patients had achieved at least a partial response (PR) after one or more courses of chemotherapy, including two with myeloma who achieved a very good partial response (VGPR). Cyclophosphamide-mobilized stem cells were collected and cryopreserved in all cases. The intensive therapy regimen for AHCT was melphalan and etoposide for patients with lymphoma (one patient also received total body irradiation) and melphalan alone (200 mg/m^2^) for patients with myeloma. Two myeloma patients also received maintenance therapy after AHCT: Patient 08 received prednisone and thalidomide and patient 09 received lenalidomide and dexamethasone.

**Table 1:** Patient characteristics pre- and post-AHCT

Patient unique identifier number	Age	Sex	Diagnosis	Treatments prior to AHCT (#)	Treatments after AHCT and before NK-92 (#)	Stage prior to NK-92
01	59	M	HL	4	2	4
02	60	F	MCL	2	0	4
03	60	M	HL	2	2	3A
04	46	M	DLBCL	3	0	4
05	46	M	DLBCL	2	5	4
06	44	M	CLL-Richter’s to DLBCL	3	1	4
07	67	M	DLBCL	2	0	4
08	59	M	MM	2	5	ISS 2
09	47	M	MM	2	2	ISS 2
10	64	F	MM	1	1	ISS 1
11	42	F	MM	1	1	ISS 1
12	66	M	MM	1	1	ISS 2

Abbreviations: AHCT – Autologous Hematopoietic Cell Transplantation, HL – Hodgkin Lymphoma, DLBCL – Diffuse Large B-Cell Lymphoma, CLL – Chronic Lymphocytic Leukemia, MM – Multiple Myeloma**,** ISS – International Staging System.

All patients exhibited evidence of disease relapse or progression after AHCT (Supplementary Table 1). The median time to relapse/progression after transplant was 20 months (range, 4–64 months). All patients had been heavily pretreated at the time of trial enrollment and had received 1–4 prior therapies prior to, and 0–5 additional therapies after, AHCT (Table 1). This included local or extended field radiation therapy in 5 cases. Median time from relapse/progression post-AHCT to receiving the first NK-92 infusion was 54 months (range, 6–128 months). Patients received a median of 3 cycles of NK-92 infusions (range, 1–6). Three patients received all 6 cycles as planned, while therapy was truncated in eight patients due to disease progression. One patient withdrew from the study after one cycle of NK-92 to receive an allogeneic hematopoietic cell transplant after a matched donor was identified.

### Toxicity

Toxicity was entirely acute infusion-related and observed in four patients who developed mild fever and/or chills (grade II maximum) that subsided with symptomatic management (Table 2). Two additional patients had either transient blurred vision (resolved within 6 hours after infusion) or nausea. One patient developed pneumonia requiring ICU admission and mechanical ventilation that was not considered related to the cell product, but led to a delay in receiving the next cycle of therapy. No delayed toxicity or graft-versus-host reactions were noted. No patient developed clinical cytokine release syndrome (CRS) of any grade*.* NK-92 infusions did not significantly affect hemoglobin, platelets, white cell count, creatinine or liver enzyme values over the duration of therapy (data not shown). Notably, there were no grade III or IV toxicities from NK-92 infusions in this study.

**Table 2:** Infusion related toxicities

Patient #	Dose level	NCI toxicity and grade					
		Fever	Chills	Nausea	Blurred vision	Fatigue	Other
01	1	0	0	0	0	0	0
02	1	0	0	1	1	0	0
03	1	0	0	1	0	1	0
04	2	0	0	0	0	0	0
05	2	2	2	0	0	0	0
06	2	1	1	0	0	0	0
07	3	1	1	0	0	0	0
08	3	0	0	0	0	0	0
09	3	0	0	0	0	0	0
10	1	0	1	0	0	0	0
11	1	0	0	0	0	0	0
12	1	0	0	0	0	0	0

### Laboratory and correlative studies

NK-92 cells prepared for each cycle of treatment were subject to quality control assessment, including flow cytometry, cytotoxicity assessment by chromium release assay, mycoplasma testing and endotoxin testing (Supplementary Table 2). NK-92 was immunophenotypically stable over multiple preparations with no contamination with endotoxin or mycoplasma detected in any batches. A subset of patients (03, 04, 05) had extensive flow cytometric analysis of peripheral blood undertaken to detect circulating NK-92 (CD56+CD71+) at various time points (Table 3). Significant numbers of NK-92 were detected in the peripheral blood of patient 03 at 15 minutes after infusion (14.9 %), but levels were undetectable at later time points. Patient 04 had background signal detected pre-infusion (0.1%), but no increase above this level at subsequent time points measured before or after subsequent NK-92 infusions. Patient 05 had a low level (0.1%) of NK-92 detected 15 minutes post infusion, but not thereafter.

**Table 3:** Flow cytometric detection of NK-92 in peripheral blood of patients

Time	% CD56+CD71+ relative to total white cell count		
	Patient 03	Patient 04	Patient 05
**Pre-infusion**	0.0	0.1	0.0
**30 min into inf.**	0.0	0.1	0.0
**15 min post**	14.9	0.1	0.1
**2 hr post**	0.1	0.0	0.0
**6 hr post**	0.0	0.0	0.0
**24 hr post**	0.0	0.0	0.0
**48 hr post**	0.0	0.0	0.0
**∼168 hr post**	0.0	0.1	0.0

Antibodies against HLA were screened in patients after each NK-92 cycle (Table 4) and compared to the HLA type of NK-92 (A 3;11, B 7;44, Cw 7;16 DR 7;15, DQ 2;6) with note made of HLA specific antibodies developing in the context of a match or mismatch. Patient 02 developed an anti-DR10 antibody detectable after cycle 1and anti-A11 antibody after cycle 2, (A11, but not DR10 is present on NK-92). Patient 05 developed anti-DR17 and anti-DR18 antibodies after cycle 2 and anti-D13 and anti-DR14 antibodies after cycle 3, none of which represented HLA antigens on NK-92.

**Table 4:** Circulating anti-HLA antibodies detected

Patient #	New persistent anti-HLA mAb present		Anti-HLA antibody relative to NK-92 cycle number*						
	Class I	Class II	Pre-infusion	1	2	3	4	5	6
01	No	No	None	None	-	-	-	-	-
02	Yes	Yes	None	DR10	A11	A11 DRB1*10	-	-	-
03	No	No	None	None	None	None	None	None	-
04	No	No	Not tested	None	-	-	-	-	-
05	No	Yes		None	DR17 DR18	DR17 DR18 DR13 DR14	-	-	-
06	No	No	**A3** **(weak)**	**A3 (weak)**	None	None	None	**A3 (weak)**	**A3 (weak)**
07	Yes	No	None	None	None	**B44** B45	**A3 A11** B13 B35 B41 **B44** B45 B47 B49 B51 B52 B53 B60 B61 B62 B72 B76 B77 B82	Not tested	-
08	No	No	None	None	-	-	-	-	-
09	No	No	None	None	None	None	-	-	-
10	Yes	No	None	None	A1 **A11 A3** A24 A80 B44 B45 B76 B82	A1 A11 A23 A24 A25 A26 A29 A30 A31 A33 A34 A36 A43 A66 A68 A69 A74 A8 B13 B27 B42 B44 B45 B49 B50 B51 B54 B55 B56 B59 B67 B7 B73 B75 B76 B78 B8 B81 B82 Cw16	A1 **A11** A23 A24 A25 A26 A29 A3 A30 A31 A33 A34 A36 A43 A66 A68 A69 A74 A80 B13 B27 B42 **B44** B45 B49 B54 B55 B56 B59 B67 B7 B73 B75 B76 B78 B8 B81 B82 **CW16**	Not tested	Not tested
11	Yes	No	None	Not tested	None	A1 B41; **B44**; B45; B47; B49; B50; B60; B76; B82	A1 **A11** A23 A24 A25 A26 A32 A36 A43 A66 B13 B37 B38 B41 **B44** B45 B47 B49 B50 B51 B52 B53 B57 B58 B59 B60 B61 B63 B76 B77 B82	A1 **A11** A23 A24 A25 A26 A29 A32 A36 A43 A66 A80 B35 B37 B38 B41 B45 B47 B50 B51 B52 B53 B57 B58 B59 B60 B61 B63 B76 B77 B82 **Cw16**	Not tested
12	Yes	No	None	B8	Not tested	Not tested	Not tested	Not tested	-

*HLA types in bold are expressed on NK-92.

**Figure 1:**
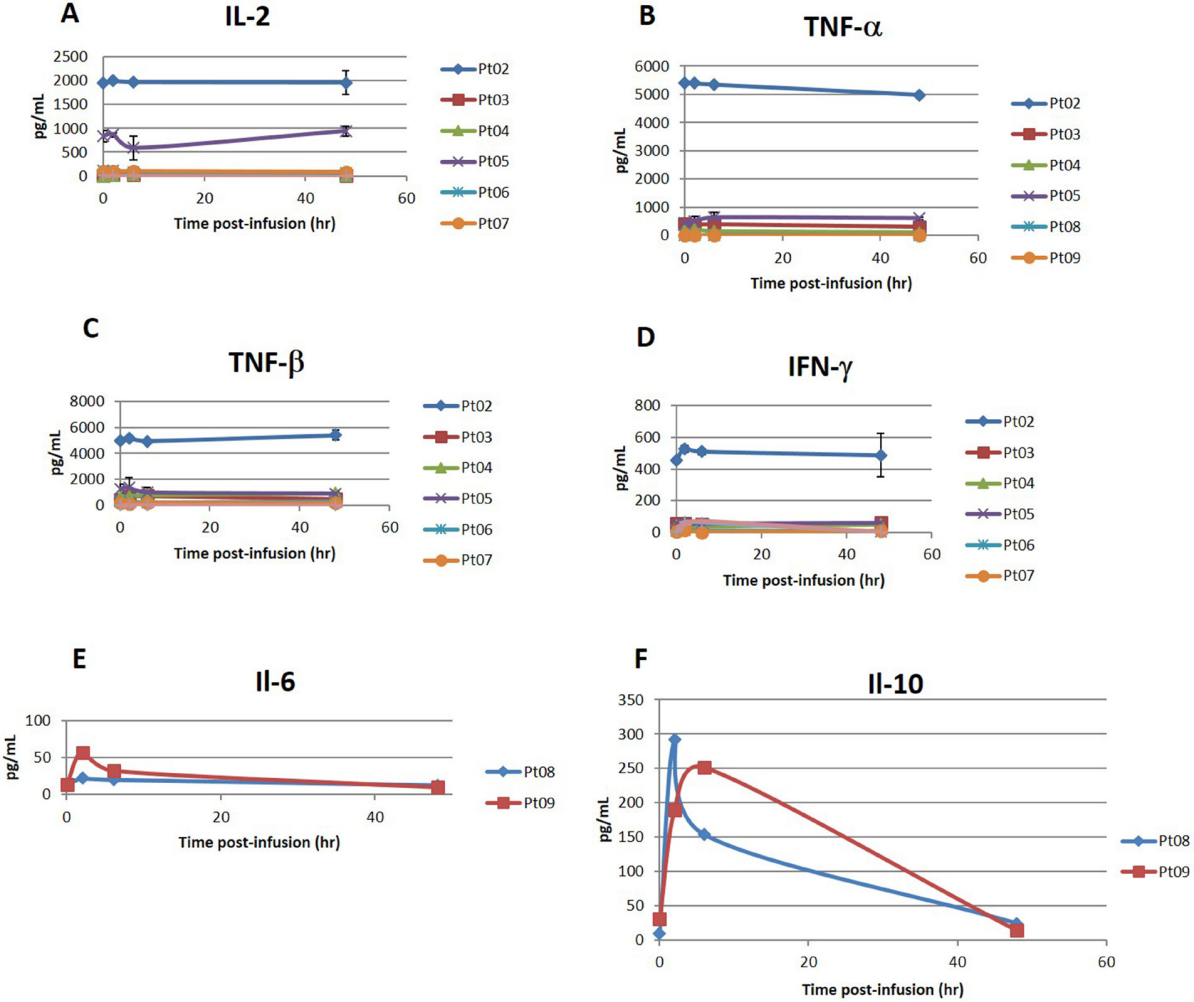
Cytokines were measured a various time points pre and post NK-92 infusions measuring: IL-2 (**A**) TNF-alpha (**B**) TNF-beta (**C**) INF-gamma (**D**) IL-6 (**E**) and IL-10 (**F**).

Patient 06 had a pre-existing anti-A3 antibody which was intermittently detectable after initiating NK-92 therapy, but the signal was weak, and did not amplify despite further NK-92 cycles. Patient 07 developed anti-B44 and anti-B45 antibodies after cycle 3,and multiple anti-A and B antibodies after cycle 4. Of these antibodies only three (B44, A3 and A11) were specific to HLA types present on NK-92. Patient 10 also developed multiple antibodies to HLA A and B types present and absent on NK-92 after cycle 2, which became more extensive after cycle 3, including anti-Cw16, specific to an NK-92 HLA type. Patient 11 developed multiple anti-HLA A and B antibodies after cycle 3, and additional antibodies were detected after cycle 4 and 5, including anti-Cw16. However, five patients with negative baseline anti-HLA antibody screens did not develop any anti-HLA antibody response to NK-92 despite receiving 1–5 cycles of NK-92. Since patient 06 had anti-A3 antibodies prior to NK-92 infusions and the signal did not amplify, a total of six patients did not mount a significant response to foreign HLA present on NK-92. In the remaining six cases with new antibodies present after NK-92 infusion, four developed antibodies directed against NK-92 HLA types and additional non-specific anti-HLA antibodies and two developed antibodies against HLA types not present on NK-92. To determine if NK-92 infusions generated a T-cell immune response, mixed lymphocyte reaction (MLR) testing was conducted after NK-92 infusion, but no T-cell proliferation was detected (Supplementary Table 3). Circulating levels of the following cytokines were measured pre- and post NK-92 infusion: IL-2, TNF-α, TNF-β, IFN-γ, IL-6 and IL-10 (Figure 1). There was no major change in cytokine levels except for a transient rise in IL-10 and IL-6 in patients 08 and 09.

### Clinical outcomes

The number of cycles of NK-92 administered ranged from 1–6 with a median of 3 cycles. Five of 12 patients exhibited a response: Patients 01 (partial), 03 (sustained CR, alive 10 years after therapy), 05 (clinical-transient), 06 (mixed-transient) and 11 (CR) (Table 5). Patient 01 had a partial response after one cycle of NK-92,underwent matched, related allogeneic bone marrow transplantation, and subsequently died of allotransplant-related complications. NK-92 therapy was discontinued in 9 patients due to progressive disease. This was based on documented clinical and radiological findings in the case of lymphoma patients, and by monitoring biochemical and immunological parameters in myeloma patients. One patient had evidence of disease progression after completing all 6 cycles of therapy. Long term remissions were documented in two patients (03 and 11), and are discussed in more detail below, and four of the 12 patients (33%) enrolled remain alive. The median survival from enrollment is 25.6 months (range, 1.1 to 127.9 months) (Supplementary Table 1).

**Table 5:** NK-92 dosing and clinical outcomes

Patient #	Dose level	Number of cycles	Number of cells administered (Total ×109)	Response	Alive
01	1	1	6	MR	No
02	1	3	15.93	PD	No
03	1	5	30	CR*	Yes
04	2	1	14.49	PD	No
05	2	3	51.84	CI**	No
06	2	6	109	MR	No
07	3	5	150	PD	No
08	3	1	27.42	PD	No
9	3	3	77.68	PD	No
10	1	6	24.67	PD	Yes
11	1	6	25.11	CR***	Yes
12	1	5	23	PD	Yes

*Patient 03 had increase in lymphadenopathy/splenomegaly interpreted as progression, with subsequent resolution to CR × 10 years after NK-92 therapy.

**Patient 05 had clinical improvement after 2 cycles of NK-92.

**Patient 11 achieved complete response after NK-92 therapy and also received concomitant Lenalidomide-Dexamethasone therapy during and after the infusions.

Abbreviations: PD: progressive disease; CR: Complete response, MR: minor response, CI: Clinical improvement in symptoms.

Patient 03 was a 60 year-old male with Hodgkin Lymphoma (nodular sclerosing subtype) who achieved remission with ABVDx6, relapsed and was given GDP salvage chemotherapy (gemcitabine, dexamethasone, cisplatin) × 2 prior to AHCT. He subsequently relapsed after AHCT, progressed on Thalidomide and Vinblastine, and then received single agent Gemcitabine. At enrollment he had clinical stage 3A disease with axillary and inguinal lymphadenopathy.

**Figure 2:**
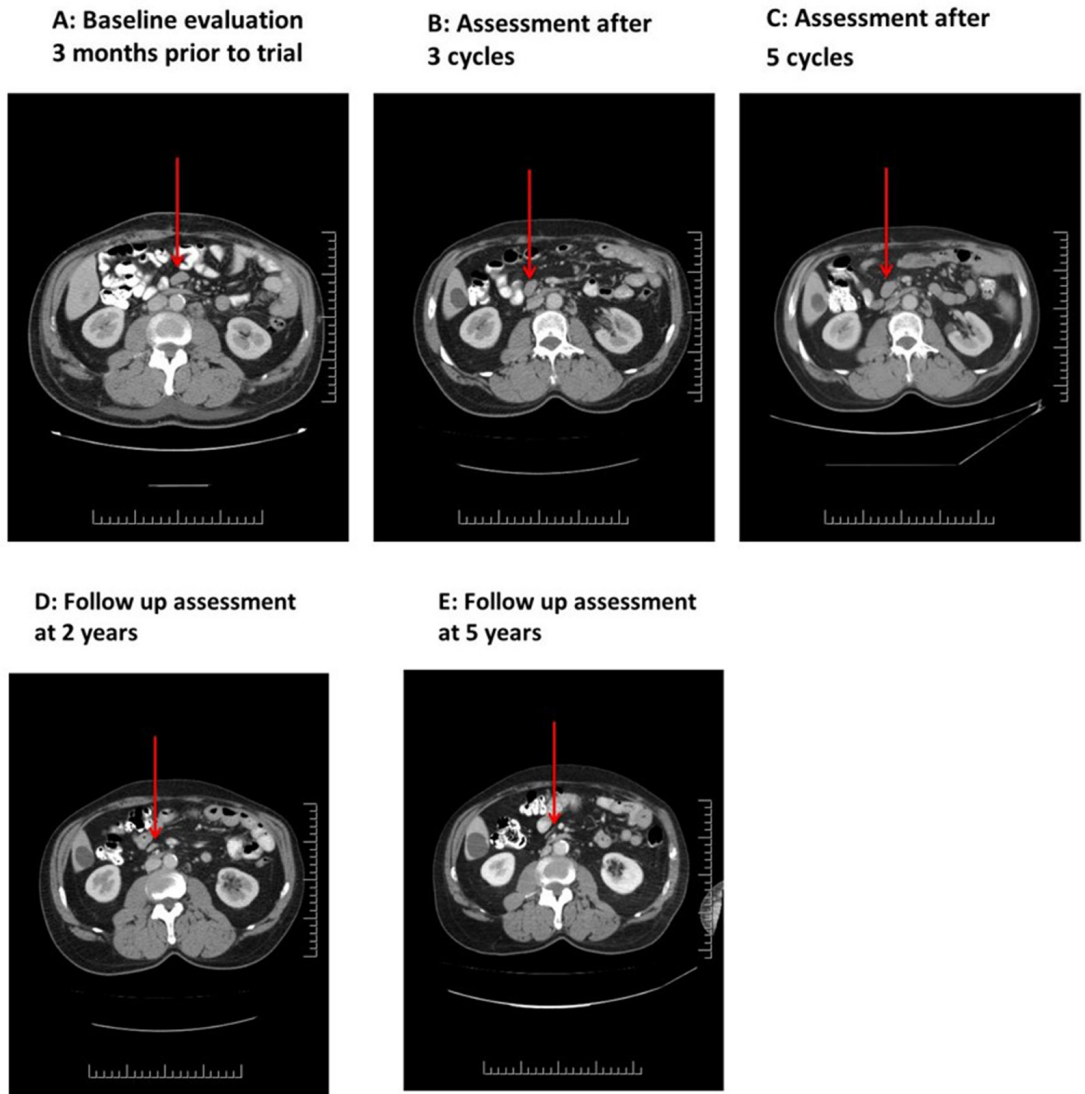
Patient 03 serial CT scans showing appearance of mesenteric lymph nodes prior to NK-92 administration (**A**) post 3- cycles of NK-92 (**B**) post 5-cycles of NK-92 therapy (**C**) and at long term follow-up 2 (**D**) and 5 (**E**) years after treatment.

**Figure 3:**
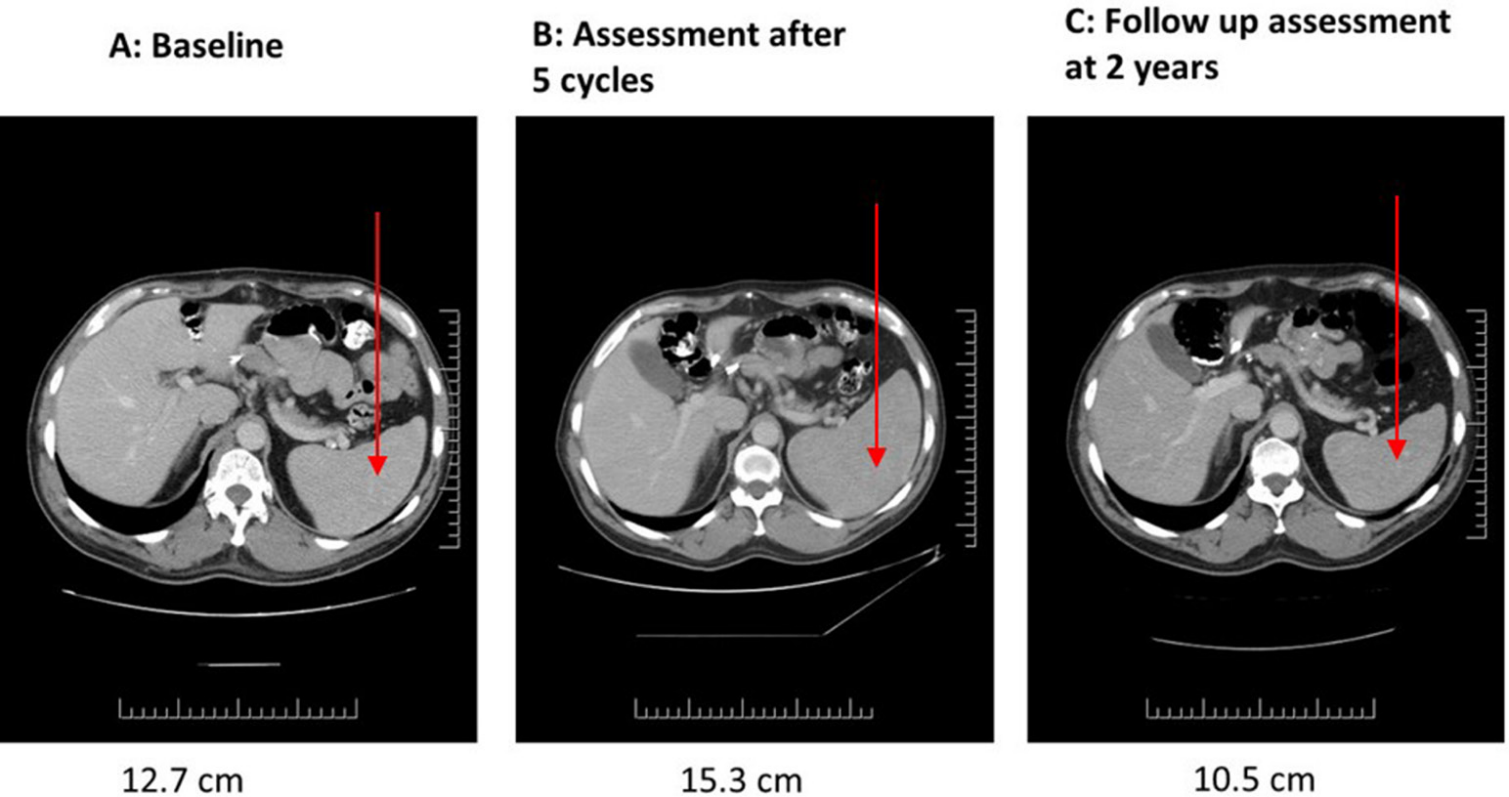
Patient 03 serial CT scans showing appearance and progression of splenomegaly at baseline (**A**, 12.7 cm), post 5 cycles of NK-92 therapy (**B**, 15.3 cm) and long-term follow-up at two years post-treatment (**C**, 10.5 cm).

A staging CT scan prior to starting NK-92 showed bilaterally enlarged axillary lymph nodes, the largest measuring 2.5 cm in its long axis, in addition to small mesenteric and para-aortic nodes (1 cm) (Figure 2A). He received a total of 5 cycles of NK-92. At interim assessment on day 23 of the first cycle, stable disease was noted with a marginal reduction in the size of the largest axillary node. Imaging at the end of 3 cycles showed a reduction in the size of some nodes but enlargement of others (Figure 2B). Imaging at the end of 5 cycles demonstrated persistence/enlargement of lymph nodes (Figure 2C) and development of splenomegaly from 12.7 cm at baseline (Figure 3A) to 15.3 cm (Figure 3B)*.* The patient was taken off study after 5 cycles due to radiological evidence of disease progression. He remained off treatment and was followed with serial CT scans that demonstrated resolution of all disease sites over the next 24 months (Figure 2D, 2E, Figure 3C). His subsequent course was complicated by anti-hypertensive drug induced thrombocytopenia which responded to prednisone and cyclosporine. He was given steroids again approximately one year later when he developed acute disseminated demyelinating encephalomyelitis secondary to cutaneous herpes zoster infection. He had complete neurological recovery following steroid therapy and a slow taper over six months. He has remained in clinical and radiological remission off any anti-cancer therapy and was asymptomatic with no detectable disease at his most recent follow up 10 years after enrollment into this trial.

Patient 11 was diagnosed with IgA kappa myeloma at the age of 37 years. Her baseline disease staging was ISS-I. She received initial chemotherapy with cyclophosphamide, bortezomib and dexamethasone × 6 cycles leading to a very good partial response (VGPR) followed by AHCT. She developed biochemical disease relapse two years later, but was managed expectantly for another 12 months after which she started salvage therapy with lenalidomide and dexamethasone (lenalidomide 25 mg × 21 days every month and dexamethasone 40 mg once a week). After four cycles of therapy she achieved a VGPR and was enrolled in the NK-92 trial and subsequently received all 6 cycles with no adverse effects. She continued therapy with lenalidomide and dexamethasone during this period, however the dose of dexamethasone was reduced to 20 mg per week (doses were omitted the week prior to and following the NK cell infusions). Serial biochemical assessment was performed after each cycle during the trial as well as six monthly afterwards. All assessments performed during and after completion of the infusions indicated CR. The patient remains on maintenance therapy with an ongoing complete response two years after treatment initiation.

## DISCUSSION

Patients with lymphoma or multiple myeloma who relapse after AHCT have a particularly poor prognosis and usually are candidates for phase I trials or receive palliative treatment. Immunotherapy has become a rapidly developing area with recent successes in treating CD19+ hematological malignancies with CAR-T cells [13–15], in the investigation of check-point blockade for melanoma, [16] lymphoma [17] and other cancers [18], and in the use of monoclonal antibodies directed against various cancers [19, 20]. Although highly effective under some circumstances, CAR-T cell therapy can exhibit considerable toxicity, including fatal cytokine release syndrome [2, 3] or encephalopathy [21], and has stimulated the investigation of other immune effector cells, including NK cells, that potentially have fewer adverse effects.

Allogeneic, KIR mismatched or haplotype NK cells can treat acute myeloid leukemia potentially more effectively than conventional transplantations [22–24]. These treatments however, face challenges that include the considerable toxicity associated with allogeneic hematopoietic transplantation in multiply treated or high risk patients, or the variable potency of autologous NK cells. In contrast, the allogeneic NK-92 line has several advantages over other NK cell therapies, including possibly lower toxicity, and more uniform product potency [8]. NK-92 expresses several activating receptors (e.g. NKp30, NKG2D and DNAM-1) and lacks expression of inhibitory killer immunoglobulin like receptors. Two prior clinical trials of NK-92 reported minimal toxicities. The first published trial of NK-92 was in refractory metastatic renal cell carcinoma (RCC) and refractory metastatic melanoma using dose escalation of up to 3 × 10^9^ for three infusions [9]. In the Arai *et al.* study infusional toxicities included one grade III fever and one grade IV hypoglycemia episode. The one metastatic melanoma patient in this trial had a minor response, while one RCC patient had a mixed response, and another survived long-term (> 4 years). A second trial of patients with solid tumors and leukemia/lymphoma employed two NK-92 infusions separated by 48 hours (maximum dose 10 × 10^9^/m^2^) [10]. In this study, one patient experienced back pain that required stopping the NK-92 infusion and giving morphine. Responses were noted in patients with lung cancer, including two mixed responses and one patient who achieved stable disease for two years. It is noteworthy that the patients in these studies received only one cycle of NK-92, consisting of 2 or 3 infusions.

Here, we have investigated a NK-92 protocol in heavily pre-treated patients with hematological malignancies, all of whom had relapsed after AHCT, to receive up to six monthly cycles of three NK-92 infusions every 48 hours per cycle in a dose escalation of 1 × 10^9^ cells/m^2^, 3 × 10^9^ cells/m^2^ and 5 × 10^9^ cells/m^2^. The dose for the final cohort was 1 × 10^9^ cells/m^2^ in view of documented responses at this lowest dose and because it was the least demanding to manufacture. Our trial also included the first patients with multiple myeloma treated with NK-92. Inclusion of the myeloma patients was of particular interest given our demonstration of the preferential cytotoxicity of NK-92 against clonogenic myeloma cells [25].

The NK-92 infusions were very well tolerated with no grade III or IV toxicities. No serious adverse events were noted and the only side effect observed, despite the high cell doses used, was mild acute infusional toxicity consisting of transient fever and/or chills in three patients. In patient 05, the infusional reaction was effectively treated with meperidine, hydrocortisone and acetaminophen. In one case (patient 02, cycle 3) blurred vision (grade I) of six hours duration was noted that resolved fully. Of the patients who had these mild infusion related reactions, one had a weak anti-HLA response to A3, one had multiple anti-class II antibodies, and another had multiple HLA-I antibodies. However, patients with the most significant pan anti-HLA response did not have fever or chills with infusions of NK-92.

Half of patients developed a significant anti-HLA antibody response, which occurred typically after the second cycle of NK-92. In the study by Arai *et al.* only two of 12 patients were evaluated for anti-HLA antibodies to NK-92 with one patient developing both anti-HLA class I and II antibodies to NK-92, with the other developing none [9]. In addition, high-resolution HLA typing for NK-92 was conducted demonstrating the following HLA type: A3, A11; B7, B44, Bw4+, Bw6+; Cw*07(3R), Cw*1601(3R); DR7, DR15; DQ2, DQ6; DR51+, DR52-, DR53+. In the study by Tonn *et al.* only one of seven patients tested positive for anti-HLA antibodies to NK-92 [10]. The increased proportion of patients exhibiting anti-HLA antibodies in our study may be related to the high cell doses administered over repeated cycles (up to six cycles or 18 infusions) of NK-92, while in the study by Tonn *et al.* patients received only one cycle, reducing the potential for immune system priming. We also note that several patients developed multiple anti-HLA antibodies beyond just HLA types present on NK-92, suggesting immunization to a common HLA epitope. However, no serious adverse effects were observed from the presence of these pan anti-HLA antibodies, and there was no correlation between development of anti-HLA antibodies and infusion reaction, although these did occur in patients with some anti-HLA antibodies, but did not recur with subsequent infusion of NK-92. Also, of note, we did not observe cytokine release syndrome in our patients.

NK-92 was detected only in one of three patients by flow cytometry at 15 minutes after infusion, suggesting rapid extravasation or sequestration of NK-92 from the circulation. Tonn *et al.* performed Y chromosome PCR on two female patients who received NK-92 infusions, and detected a signal at 5 minutes (0.1%), 1 hour (0.01%) and beyond (0.001%–0.01%) at very low levels after infusion. This appears to be consistent with our flow cytometry data.

Patients 01 to 07 had lymphomas of different subtypes (Table 1) and were heavily pretreated in many cases for multiply relapsed chemotherapy-resistant disease. One patient nonetheless experienced a minor response (01), another a mixed response (06), and one patient with relapsed Hodgkin lymphoma (03) remains in complete remission 10 years after receiving NK-92. Of the five multiple myeloma patients treated with NK-92, three remain alive. One patient (11), achieved a complete response, but also received concomitant standard maintenance therapy with lenalidomide and dexamethasone.

In summary, we have shown that high dose infusions of NK-92 are safe, have minimal toxicity and in heavily pre-treated patients with blood cancers, show preliminary evidence of tumour responses and the potential for a complete remission. This study infused the highest reported doses of NK-92, including up to 6 cycles with a maximum of 18 infusions over a six month period and a total dose of up to 150 × 10^9^ cells.

NK-92 has subsequently been genetically modified to express high affinity CD16 and IL-2 (termed haNK), making it capable of antibody dependent cell-mediated cytotoxicity and proliferation *in vitro* in the absence of IL-2. The FDA recently approved haNK cells for experimental use in humans. The haNK platform can be readily combined with existing or novel humanized monoclonal antibodies to target cells bearing tumour-associated antigens and provides a means of systematically testing a variety of cancers.

## MATERIALS AND METHODS

### Patient eligibility

The study (NCT00990717) was conducted at the Princess Margaret Cancer Centre (University Health Network, [UHN]: protocol CTP04.NK92.01), Toronto, Canada under Health Canada (Parental control number 096422- file#9427-P1648–55C, last amendment control number 164230) and UHN institutional research ethics board approval (REB# 03-0018-C). Study inclusion criteria included: i). hematological malignancy in relapse after an autologous hematopoietic cell transplant; ii). measurable disease; iii). Adequate function of bone marrow (granulocytes >= 0.5 × 10^9^/L, platelets >= 50 × 10^9^/L), kidneys (Cr <= 1.5 × upper limit of normal (ULN), Ca <= 1.25 × ULN) and liver (Bilirubin <= 1.5 × ULN, AST/ALT <= 3 × ULN) at the time of cell infusion (; iv) Life expectancy of at least 12 weeks. V). adult > 18 years age; vi). ECOG performance status of 0–2. Exclusion criteria were: i). Pregnant or nursing female. ii). Concurrent treatment within 28 days with other experimental drugs or anticancer therapy. Exceptions were patients who received radiation therapy less than 4 weeks before study provided the volume of bone marrow treated was less than 10%; hydroxyurea for high white cell counts less than 48 hours prior to study; and for patients with multiple myeloma on maintenance chemotherapy with lenalidomide and dexamethasone. A protocol amendment was made to include maintenance chemotherapy for all eligible patients relapsing after auto-transplantation. In order to circumvent the effect of steroids on the cell therapy product, maintenance therapy in eligible multiple myeloma patients was modified to omit dexamethasone one week prior to and after the NK cell infusion and to reduce the dose to 20 mg per week instead of 40 mg in other periods. iii). known HIV, HBV or HCV infection; and iv) central nervous system involvement.

### Trial design

The trial was a single-center, non-randomized, non-blinded, open-label, dose-escalation study. Three patients were treated at each dose level: 1 × 10^9^ cells/m^2^, 3 × 10^9^ cells/m^2^ and 5 × 10^9^ cells/m^2^. Treatment consisted of up to 6 cycles at 28 day intervals of three infusions each of the cell dose on days 1, 3 and 5. For the last three patients, a dose level would be chosen based on toxicity, clinical response and feasibility of cell manufacturing.

### Manufacture of NK-92 cells

NK-92 was obtained from ConkWest (Del Mar, CA, USA). NK-92 Master Cell Bank production and testing was performed by BioReliance Corporation (Rockville, MD, USA) according to current good manufacturing practice (cGMP) guidelines and regulations using NK-92 stock cultures tested for sterility and mycoplasma. NK-92 cells were expanded, stored in liquid nitrogen to create a working cell bank. NK-92 cryovials were then retrieved as required, and expanded in GM1 medium. GM1 medium consisted of 0.2 µm filtered X-Vivo 10 medium (Lonza, Basel, Switzerland) supplemented with 2.5% human AB serum (Lonza), 450 U/mL Proleukin recombinant human (rh)IL-2 (Novartis, Basel, Switzerland), 0.6 mM L-asparagine (Sigma, St Louis, MO, USA), 3 mM L-glutamine (Life Technologies, Carlsbad, CA, USA) and 1.8 mM L-serine (Sigma).

Manufacture of clinical-grade NK-92 was performed under GMP conditions at the Philip S. Orsino Cell Processing Facility at Princess Margaret Hospital in Toronto, Ontario, Canada. Three weeks before the targeted date of infusion, NK-92 cell cultures were initiated from the NK-92 Working Cell Bank. Cells were expanded in GM1 medium. The cultures were initiated at 2.5 × 10^5^ cells/mL in 25 mL (6.25 × 10^6^ cells) in 1.6 L VueLife culture bags (American Fluoroseal Corp., Gaithersburg, MD, USA), with the addition of GM1 medium every 3 days, maintaining a density of approximately 2.5 × 10^5^ cells/mL, and with mild disruption of cell aggregates. Final yields of approximately 0.8 × 10^9^ cells per culture bag were achieved after 21 days, with more than 70% viability. The final feeding with (rh)IL-2 and fresh medium was 48 h before the first day of infusion of the expanded NK-92 product.

The NK-92 product was required to pass release testing prior to infusion into the patient. As this product was harvested and infused on the same day, full sterility results were not available and to ensure patient safety, a stat Gram stain was performed immediately before infusion to detect any gross contamination. Samples were also taken for endotoxin testing with release criteria of < 0.5 EU/ml (Lonza) and mycoplasma testing (WuXi AppTec, Philadelphia, PA, USA). NK-92 cell batches were required to have viability of 70% or greater for release. The final NK-92 cell product was re-suspended in GM2 medium (Plasma-Lyte-A medium (Baxter, Deerfield, IL, USA) supplemented with 2.5% human AB plasma (SeraCare, Milford, MA, USA), irradiated with 10 Gy (using ^137^Cesium source irradiator) and infused fresh to the patient.

### Treatment with NK-92 and follow-up

On the day of infusion, hydration (200 mL of normal saline per hour) was given to the patient 2 h prior to NK-92 infusion and continued for 2 h after infusion. The total volume of NK-92 cell infusate was 100 to 300 mL,depending on dose and body surface area. Cells were infused at a rate of 5 mL/min, with a total infusion time of approximately 20 to 60 min. Allopurinol (300 mg) was given once daily by mouth for 5 days starting on the first day of infusion. All patients received pre-medication with Acetaminophen (750 mg PO) and Diphenhydramine (25–50 mg IV) before the start of each cell infusion.

Complete tumor staging was performed prior to NK-92 treatment. During cell infusion, patients were closely monitored, with vital signs recorded at 0, 15, 30, 60, 90, 120 and 240 min and every 24 h thereafter until day 5. Patients were admitted overnight after the first infusion and then followed on an outpatient basis after subsequent infusions. Hematology (CBC, differential and platelets) and biochemistry (BUN, serum creatinine, electrolytes, bilirubin, alkaline phosphatase, AST, ALT, LDH, serum protein, calcium, albumin and glucose) were performed on Day 3, 5, 7, 14 and 28 for the first cycle and on the first day of each cycle. X-ray or CT scans were repeated at 4 weeks after the treatment course to assess disease response, and thereafter, according to clinical indication. Coagulation (PT and/or INR, PTT) and urinalysis were repeated on the first day of each cycle. Patients were examined daily for clinical toxicity from NK-92 infusion for the first 7 days and then weekly thereafter until 4 weeks after cell infusion. NCI-CTC version 3 criteria were used to document toxicities. Tumor response was assessed according to Response Evaluation Criteria in Solid Tumors (RECIST).[26] Additionally, a minor response was defined as regression of target tumor lesions by 10–30% with no new lesions and no non-target lesion progression.

### NK-92 functional assays and biological correlative studies

#### Immunophenotypic analysis

Immunophenotypic analysis by flow cytometry was done to ensure that the product was consistent for each infusion and to assess the functional capacity of the *ex vivo*-expanded NK-92 cells. NK-92 cells were tested for the presence of the following markers: CD54, CD11A, CD45, CD16, CD23, CD20, CD4, CD8, CD3, CD56, CD2, CD7, CD1A, CD28, CD34, CD10, CD14 and CD71 and confirmed to be negative for CD16, CD23, CD20, CD4, CD8, CD3, CD1A, CD34, CD10 and CD14. (BD Biosciences, San Jose, CA, USA). Prior to each cycle the NK-92 product was tested for a subset of the above markers with the exception of CD71, which was used in assays to detect NK-92 in the peripheral blood of patients after infusion.

### Chromium release assay

To assess potency prior to infusion, the NK-92 product was assayed for cytotoxicity against the K562 cell line using the chromium release assay using a range of effector to target ratios (50:1, 40:1, 20:1, 10:1 or 5:1). K562 was obtained from American Type Cultural Collection (ATCC, Manassas, VA, USA) and maintained in RPMI (Invitrogen, Grand Island, NY, USA) supplemented with 10% FBS.

Target cells were washed in 10 mL AIM-V serum-free medium (Life Technologies) and 1 × 10^6^ cells were re-suspended in 100 µL CiNa_2_^51^CrO_4_ (100 µCi) for 2 h prior to treatment, washed three times and re-suspended in AIM-V medium prior to treatment with NK-92. Five thousand radiolabeled K562 cells were added to individual wells of a 96-well U-bottomed plate. NK-92 at various concentrations was added to K-562 to yield 50:1, 40:1, 20:1, 10:1 or 5:1 effector to target (E:T) ratios and plates were incubated at 37°C, 5% CO_2_ for 4 h. Plates were centrifuged at 400 rpm for 5 min; 100 µL supernatant was collected from each well and transferred into collection tubes. The amount of ^51^Cr present in supernatants was determined using a gamma-counter. Percentage lysis was calculated using the formula % lysis equals to (E – S) × 100/(M – S), where E is the ^51^Cr -release from an experimental sample, S the spontaneous release in the presence of complete IMDM medium and M the maximum release upon cell lysis with Triton X-100 10%.

### Biological correlative studies

#### Anti NK-92 humoral immune response

Anti-HLA antibodies were also assessed for one week prior to treatment and after each cycle of NK-92. Antibody testing was conducted by the UHN HLA laboratory.

### Kinetics of NK-92 cells

Patient peripheral blood samples were tested to detect the presence or absence of NK-92 cells as measured by flow cytometry using CD56 and CD71 as the markers (BD Biosciences). CD71 is not detected on primary NK cells unless activated and is constitutively expressed on NK-92. Using this approach we established a flow cytometry based assay that could detect NK-92 from mixtures of total white cells and NK-92 with a 1:1000 detection limit. NK-92 in select patients’ peripheral blood was measured at the following time points: pre-infusion, 15 min into infusion, 30 min, 2 hours, 6 hours, 24 hours, 28 hours, and 168 hours post-infusion.

### Cytokine assays

Patient sera were tested for cytokine levels of IL-2,IFN-γ, TNF-α, IL-6, IL-10 and TNF-β as measured by enzyme-linked immunosorbent assay (ELISA) at the following time-points: pre-infusion, 2 hours, 6 hours, and 48 hours post-infusion.

### Mixed lymphocyte reaction

Patient PBMCs were isolated using Ficoll (Sigma) and evaluated in a mixed lymphocyte reaction (MLR) assay for specific lymphocyte (CTL) activation against NK-92 one week prior treatment, on day of treatment and two weeks after treatment. Testing of MLR was repeated every cycle. Briefly, irradiated stimulator cells (NK-92, THP-1 or PBMCs from a healthy donor) were mixed with responder cells (patient’s PBMCs or PBMCs from a different healthy donor) in RPMI 1640 medium supplemented with 10% fetal bovine serum. Culture medium was negative control and PHA (10 µg/mL) the positive control. Cultures were pulsed at days 4 and 5 with 1 µCi [^3^H]-thymidine per well, incubated overnight at 37°C and 5% CO_2_ and harvested for counting. Thymidine incorporation was measured by a scintillation counter (TopCount, Perkin Elmer Life Science, Inc.).

## SUPPLEMENTARY MATERIALS TABLES



## Abbreviations

ABVD: Adriamycin (doxorubicin), bleomycin, vinblastine, dacarbazine
AHCT: autologous hematopoietic stem cell transplantation
cGMP: current good manufacturing practice
CI: clinical improvement
CR: complete response
CLL: chronic lymphocytic leukemia
DLBCL: diffuse large B-cell lymphoma
GDP: gemcitabine, dexamethasone, Platinol (cisplatin)
GMP: good manufacturing practice
haNK: high affinity NK
HCT: hematopoietic stem cell transplantation
HL: Hodgkin lymphoma
HLA: human leukocyte antigen
ISS: International staging system
MCL: mantle cell lymphoma
MLR: mixed lymphocyte reaction
MM: multiple myeloma
NCI-CTC: National Cancer Institute- common terminology criteria
PR: partial response
RCC: renal cell carcinoma
RECIST: response evaluation criteria in solid tumors
ULN: Upper limit of normal
VGPR: Very good partial response
