# Supplementary material for: A phase I trial of NK-92 cells for refractory hematological malignancies relapsing after autologous hematopoietic cell transplantation shows safety and evidence of efficacy
Source: Oncotarget. 2017 Jul 12;8(51):89256–68. doi: 10.18632/oncotarget.19204 (PMC5687687; doi:10.18632/oncotarget.19204)
Supplement: Supplementary file 1 [file oncotarget-08-89256-s001.pdf]

A phase I trial of NK-92 cells for refractory hematological malignancies relapsing after autologous hematopoietic cell transplantation shows safety and evidence of efficacy

SUPPLEMENTARY MATERIALS

Supplementary Table 1: Time from relapse post AHCT to initiation of NK-92 trial and survival

Patient Number	Duration of response post AHCT (months)	Time from AHCT to NK92 (months)	Time from Relapse to NK92 (months)	Survival from trial enrolment (months)
01	33.7	69.7	36.0	72.1
02	34.5	37.7	3.2	24.7
03	9.5	52.2	42.8	127.9
04	4.1	6.0	1.9	7.2
05	18.8	68.2	49.4	12.1
06	20.6	30.5	9.9	17.6
07	64.1	66.7	2.6	36.5
08	3.9	126.0	122.0	1.1
09	18.7	89.8	71.1	3.0
10	25.9	33.7	7.8	44.1
11	23.0	49.7	26.7	37.0
12	16.1	55.6	39.5	26.4

Supplementary Table 2: Quality control testing on NK-92 on patient 01 first cycle

Parameter	Result
Gram stain	negative
Mycoplasma	negative
Endotoxin	negative
Flow cytometry (positive)	CD 2, 7, 11A, 28, 45, 54, 56
Flow cytometry (negative)	CD 1A, 3, 4, 8, 10, 14, 16, 20, 23, 34
Cytotoxicity (% lysis at E:T ratio)	5:1 (32 +/–3), 10:1(39 +/–2), 20:1 (49 +/–5)

Supplementary Table 3: Summary of mixed lymphocyte reaction assays of patient lymphocytes mixed with NK-92

Patient[#]	MLR tested	Number of tests*	MLR results
01	Yes	1	Negative
02	Yes	2	Negative
03	Yes	5	Negative
04	Yes	1	Negative
05	Yes	3	Negative
06	Yes	6	Negative
07	Yes	4	Negative
08	No	N/A	N/A
09	Yes	2	Negative
10	Yes	6	Negative
11	Yes	5	Negative
12	No	N/A	N/A

*Patients had MLR testing from pre-cycle 2, up to post-treatment in some cases.
